# Longitudinal impact of COVID-19 pandemic on mental health of children in the ABCD study cohort

**DOI:** 10.1038/s41598-022-22694-z

**Published:** 2022-11-15

**Authors:** Sayo Hamatani, Daiki Hiraoka, Kai Makita, Akemi Tomoda, Yoshifumi Mizuno

**Affiliations:** 1 Research Center for Child Mental Development, University of Fukui, Fukui, Japan; 2 Division of Developmental Higher Brain Functions, United Graduate School of Child Development, University of Fukui, Fukui, Japan; 3 Department of Child and Adolescent Psychological Medicine, University of Fukui Hospital, Fukui, Japan; 4 Research Center for Child Mental Development, University of Chiba, Chiba, Japan; 5 Japan Society for Promotion of Science, Tokyo, Japan

**Keywords:** Paediatrics, Public health, Paediatric research

## Abstract

A large longitudinal study on the impact of the COVID-19 pandemic on mental health in children is limited. This large-scale longitudinal observational study examines the pandemic’s effects on children’s mental health while considering the effects of parental care styles. The Adolescent Brain Cognitive Development Study is a large-scale, longitudinal multicenter study in the United States. Of the 11,875 children aged 9–12 years in its database, 4702 subjects were selected for this study. The child behavior checklist and parental monitoring questionnaire (PMQ) were used to assess children’s mental health and parental support styles, respectively. Data collected before and during the pandemic were compared. Withdrawn/depressed and attention problems significantly worsened during compared to before the COVID-19 pandemic (*p* < 0.001, withdrawn/depressed; 53.4 ± 5.7 to 53.7 ± 5.9, attention problems; 53.4 ± 5.4 to 53.6 ± 5.6). However, the T scores are in the normal range both before and during the crisis. Simple slope analysis found withdrawn/depressed problems and aggressive behavior worsened when the PMQ was 1 SD below the mean, and rule-breaking behavior was improved when the PMQ was 1 SD above the mean. While the COVID-19 pandemic exacerbated children’s depressive symptoms and attention issues, the effects may be minor. Additionally, parental involvement serve as a protective factor for the child’s mental health even during the pandemic.

## Introduction

The Coronavirus 2019 (COVID-19) pandemic originated in late 2019^1^. According to the World Health Organization (WHO), as of December 14, 2021, more than 260 million people have been infected and more than 53 million have died due to the virus^2^. The United States accounted for about one in five COVID-19 infections worldwide, and the number of children infected in the United States has climbed to about 7.2 million in December 9 2021^3^. Globally, people have incorporated measures in their daily lives to curb the spread of COVID-19^4^. The situation raises concerns over the virus’s impact on mental health^5^. The percentage of children who have visited the emergency department in hospitals due to mental health problems has increased by 31–50% in the United States since the onset of the pandemic^6,7^. School closures to maintain social distancing norms can disrupt children’s physical activities and social interactions, and affect their mental health.

A meta-analysis covering 29 studies showed that the percentage of children who experienced high clinical anxiety and depression increased to about 20–25%, doubling from the pre-pandemic numbers^8^. However, most of these studies were cross-sectional; a longitudinal study is required to investigate the COVID-19 pandemic’s effects on children’s mental health. Indeed, the few longitudinal investigations conducted have reported increased mental health problems such as depression and anxiety^9–12^. Conversely, a longitudinal study in southwest England reported an overall reduced risk of anxiety, no significant changes in the risk of depression, and enhanced wellbeing during the pandemic^13^. Thus, the results of previous longitudinal studies that included pre-pandemic data are inconsistent; it is unclear whether the COVID-19 pandemic has a negative effect on the mental health of children. Hence, it is necessary to additionally investigate the impact of the pandemic on children’s mental health using large longitudinal data.

Additionally, parental involvement affects the overall mental health of the child^14^ and even has a lasting effect on the development of the child’s personality and other psychological characteristics^15,16^. A proper parenting style strengthens family ties and meets the child’s psychological needs^17^. In addition, a proper parenting style reduces children's clinical symptoms and behavioral problems^18,19^. In particular, perceived family connectedness is likely to have a protective effect on a child’s mental health during traumatic events such as disasters^20^. Therefore, in a long-term public health crisis, such as the COVID-19 pandemic, parent–child connections appear to be crucial for children’s mental health. Moreover, as quarantine measures continue, children inevitably spend more time at home with their parents and it is worth investigating to what extent parental involvement affects a child’s mental health during the pandemic. Although an online cross-sectional study in China has reported that parent–child communication is important for children’s mental or behavioral health during the pandemic^21^, there is limited evidence of the impact of the frequency of such communication and parent–child involvement on a child’s mental health.

Given this gap, we used samples from the large, longitudinal Adolescent Brain Cognitive Development (ABCD) Study to investigate the mental health of children before and during the COVID-19 pandemic. The largest long-term study of brain development and child health in the United States, the ABCD Study has targeted more than 10,000 children aged 9–10 since 2015^22^. By using the data from this study, it is possible to infer large-scale longitudinal data, including pre-pandemic data. Therefore, the purpose of this study is two-fold: First, to test the hypothesis that children’s mental health has worsened during the COVID-19 pandemic compared to pre-pandemic times; second, to test the hypothesis that parental involvement serves as a protective factor for children’s mental health during the pandemic.

## Method

### Participants

**Figure 1:**
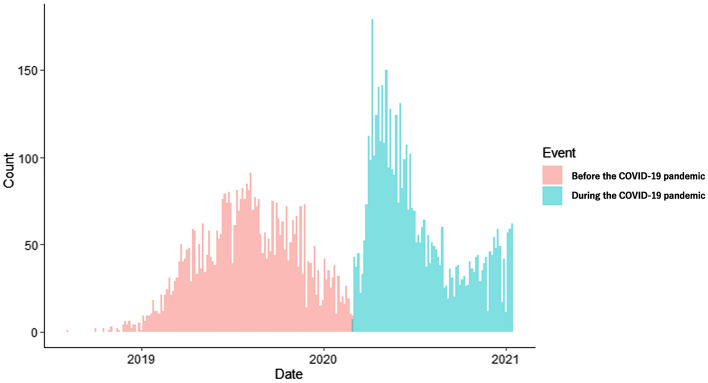
Distribution of samples before and during the Covid-19 pandemic.

Participants selected for the present study were already enrolled in the ongoing Adolescent Brain Cognitive Development (ABCD) Study. A sample of 11,875 children aged 9–10 enrolled at 21 study sites across the United States^22^. Of these, a group who have CBCL data after the beginning of the COVID-19 pandemic (March 1, 2020) in the third-year follow-up data in the ABCD Study were defined as “during the COVID-19 pandemic” (4885 children), and a group who had CBCL data before the COVID-19 pandemic in the second-year follow-up data in the ABCD Study, were defined as “before the COVID-19 pandemic” (4702 children) in this study. The data before the COVID-19 pandemic were collected from August 4, 2018, to February 29, 2020, and the data during the COVID-19 pandemic were collected from March 1, 2020, to January 15, 2021 (Fig. 1). We have included a flowchart in the Supplementary Materials to illustrate the process. The data was sourced from the NIMH Data Archive website (https://data-archive.nimh.nih.gov/abcd). The present study mainly used data from the ABCD 4.0 data release (http://dx.doi.org/10.15154/1523041). Full recruitment details of the ABCD Study are described in the literature^23^. All parents provided written informed consent and all children provided assent. All procedures complied with the Declaration of Helsinki. The research protocol was approved by the Ethics Review Committee of the University of Fukui (Assurance No. FU-20210067). This study followed the Strengthening the Reporting of Observational Studies in Epidemiology (STROBE) reporting guideline.

### Measures

#### Demographic variables and additional covariates

The following covariates were dummy coded: race/ethnicity (White, Black, Hispanic, Asian, and other), twin or triplet status, and sex. Annual household income was treated as a 5-level categorical variable. Based on a previous study^24^, the first 5 of the 10 household income levels were collectively assigned a value of 1 (i.e., < $50,000). The subsequent categories used were coded as 2 ($50,000–$74,999), 3 ($75,000–$99,999), 4 ($100,000–$199,999), and 5 ($200,000 or more), respectively (see Table 1). Moreover, parental educational level was included as a continuous covariate in terms of years of study. It was recoded as follows: 12th grade, high school, and general education development = 12 years; some college and associate degree = 14 years; bachelor’s degree = 16 years; master’s degree = 18 years; professional and doctoral degrees = 20 years.

**Table 1:** Demographics and study sample.

Characteristic		
	*M* ± *SD* *N* = 4702	*M* ± *SD* *N* = 4885
Child age (month)	143.5 ± 7.8	154.5 ± 7.8
**Education (years)**		
Mother	15.5 ± 2.5	15.5 ± 2.5
Father	15.3 ± 2.8	15.3 ± 2.8
		*N* (%)
**Sex**		
Male	2477 (53%)	2576 (53%)
Female	2225 (47%)	2309 (47%)
**Race**		
White	2731 (58%)	2817 (58%)
Hispanic	899 (19%)	947 (19%)
Black	495 (11%)	518 (11%)
Asian	110 (2.3%)	117 (2.4%)
**Income**		
< $50,000	1062 (24%)	1113 (24%)
$50,000–74,999	587 (13%)	613 (13%)
$75,000–99,999	676 (15%)	695 (15%)
$100,000–199,999	1479 (34%)	1523 (33%)
≥ $200,000	584 (13%)	611 (13%)

*M* mean, *SD* standard deviation.

To assess the mental health of children before and during the pandemic, we used the Child Behavior Checklist (CBCL) (file name: abcd_cbcls01). Similarly, we used the third-year the Parental Monitoring Questionnaire (PMQ) data to assess parental involvement during the pandemic.

#### Child Behavior Checklist (CBCL)

The Child Behavior Checklist (CBCL)^25,26^ is a questionnaire for parents to assess behavioral and emotional problems of children. The CBCL consists of 113 questions that measure aspects of children’s behavior over six months and is scored on a 3-point Likert scale (0 = not true; 1 = somewhat or sometimes true; 2 = very true or often true). The CBCL comprises a total score, two broad-band scales (internalizing and externalizing problems), and eight syndrome scales (anxious/depressed, withdrawn/depressed, somatic complaints, social problems, thought problems, attention problems, aggressive behavior, and rule-breaking behavior). The higher the score, the greater the problem. All scores were recorded as *t*-scores and utilized. *T*-scores ⩾ 70 on the eight syndrome scales and ⩾ 64 on the internalizing, externalizing, and total problems scales indicates placement in the clinical range. *T*-scores in the borderline range (65–69 on the eight syndrome scales, 60–63 on the internalizing, externalizing, and total problems scales) suggest cause for concern. Scores below this level are in the normal range^26^.

#### Parental Monitoring Questionnaire (PMQ)

The Parental Monitoring Questionnaire (PMQ)^27^ is a self-administered, 5-item questionnaire for children to measure parental monitoring and supervision. The PMQ consists of the following categories: parental monitoring of location; parental monitoring of who their children spend time with; parent and child contact; child disclosure; and parental monitoring via family dinner frequency. The range of all items was 1–5 (1 = Never; 2 = Almost Never; 3 = Sometimes; 4 = Often; 5 = Always or Almost Always). An average score was calculated based on all five items; the higher the score, the higher the frequency of the parents’ monitoring behavior.

#### Statistical analyses

All analyses were performed using R version 4.1.1^28^. The effects of the pandemic were analyzed by comparing the data obtained before and during the COVID-19 pandemic. Models for the CBCL outcome were estimated using a linear mixed-effects design using the ImerTest package^29^ after centering the independent variable with the average value. Key predictors for each model included PMQ, time, and PMQ × time. Time was recoded as follows: before the COVID-19 pandemic = −0.5, during the COVID-19 pandemic = 0.5. Covariates included age at before the COVID-19 Pandemic, sex, parental education, household income, race, twin, and triplet, while random intercepts details included individual, sibling, and location information (file name: abcd_lt01). Furthermore, to understand the characteristics of the interaction, a simple slope analysis was performed on the variables for which interactions showed significant differences pre- and during the pandemic (withdrawn/depressed, rule-breaking, and aggressive behaviors). Similarly, simple slope analysis were performed for each sub-item of PMQ as well. Additionally, a linear mixed-effects analysis was performed for the groups that did and did not exceed the boundary area of the *T*-score before the pandemic, respectively, to investigate whether the change in score differs depending on the level of CBCL. Lastly, to examine the impact of attrition, baseline data were compared using the Welch Two Sample *t*-test with and without data up to the third year. All *p* values were two-tailed, and the results were deemed statistically significant at *p* < 0.05 (Bonferroni-corrected). We removed the data of participants who were missing one or more variables.

## Results

Data of 4702 and 4885 children were obtained from before and during the COVID-19 pandemic. Characteristics of these subjects are provided in Table 1. The average values of CBCL and PMQ before and during the COVID-19 pandemic are shown in Table 2.

**Table 2:** Child behavior checklist and parent–child relationships before and during the COVID-19 pandemic.

CBCL (*T*-score)	Before COVID-19 pandemic	During COVID-19 pandemic
	*N* = 4702	*N* = 4885
	*Mean* ± *SD*	*Mean* ± *SD*
Total score	44.8 ± 11.0	44.9 ± 11.3
Internalizing problem	47.8 ± 10.4	47.9 ± 10.5
Externalizing problem	44.3 ± 9.6	44.4 ± 9.5
Withdrawn/depressed	53.4 ± 5.7	53.7 ± 5.9
Somatic complaints	54.6 ± 5.8	54.4 ± 5.8
Social problems	52.6 ± 4.7	52.6 ± 4.8
Thought problems	53.6 ± 5.7	53.6 ± 5.7
Attention problems	53.4 ± 5.4	53.6 ± 5.6
Rule-breaking behavior	51.9 ± 3.9	51.9 ± 3.8
Aggressive behavior	52.3 ± 4.8	52.3 ± 4.8
Anxious/depressed	53.2 ± 5.8	53.3 ± 6.0
PMQ	–	4.40 ± 0.49

*M* mean, *SD* standard deviation, *CBCL* child behavior checklist, *PMQ* parental monitoring questionnaire.

Regarding the CBCL, the main effects of time were significant after multiple testing correction: withdrawn/depressed (*p* < 0.001) and attention problems (*p* < 0.001). Except for somatic complaints, the main effects of PMQ were significant after multiple testing correction; anxious/depressed (*p* < 0.05) and social problems (*p* < 0.01) were significant and all others (i.e., internalizing problem, externalizing problem, withdrawn/depressed, thought problems, attention problems, rule-breaking behavior, and aggressive behavior) were significant at below 0.1% levels (*p* < 0.001). In addition, the interactions between parent monitoring behavior and time point of the withdrawn/depressed, rule-breaking behaviors, and aggressive behaviors were significant; however, after multiple testing correction, significant difference was observed in only withdrawn/depressed (see Table 3). Table S1 to Table S11 in the supplementary materials show full results.

**Table 3:** Effects of parent monitoring and time point on mental health in children.

Outcome	Variable	B	95%CI	*R*^*2*^	*p* value	Bonferroni corrected *p* value	β
**CBCL**							
Total score	Time	0.208	− 0.043 to 0.459	0.050	0.105	1.000	0.019
	PMQ	− 2.636	− 3.297 to − 1.975		< 0.001***	< 0.001***	− 0.237
	Time: PMQ	− 0.213	− 0.749 to 0.322		0.435	1.000	− 0.019
Internalizing problem	Time	0.139	− 0.132 to 0.411	0.040	0.315	1.000	0.013
	PMQ	− 1.872	− 2.510 to − 1.234		< 0.001***	< 0.001***	− 0.179
	Time: PMQ	− 0.176	− 0.755 to 0.404		0.553	1.000	− 0.017
Externalizing problem	Time	0.251	0.020 to 0.481	0.036	0.033	0.362	0.026
	PMQ	− 2.206	− 2.785 to − 1.627		< 0.001***	< 0.001***	− 0.231
	Time: PMQ	− 0.396	− 0.887 to 0.095		0.114	1.000	− 0.041
Withdrawn/depressed	Time	0.370	0.203 to 0.537	0.040	< 0.001***	< 0.001***	0.064
	PMQ	− 1.669	− 2.021 to − 1.317		< 0.001***	< 0.001***	− 0.290
	Time: PMQ	− 0.518	− 0.874 to − 0.162		0.004**	0.048*	− 0.090
Somatic complaints	Time	− 0.132	− 0.309 to 0.044	0.029	0.142	1.000	− 0.023
	PMQ	− 0.346	− 0.682 to − 0.010		0.044*	0.483	− 0.060
	Time: PMQ	− 0.088	− 0.464 to 0.289		0.648	1.000	− 0.015
Social problems	Time	− 0.002	− 0.128 to 0.124	0.023	0.976	1.000	− 0.000
	PMQ	− 0.611	− 0.893 to − 0.329		< 0.001***	0.002**	− 0.129
	Time: PMQ	0.043	− 0.226 to 0.311		0.754	1.000	0.009
Thought problems	Time	− 0.012	− 0.166 to 0.141	0.027	0.875	1.000	− 0.002
	PMQ	− 0.925	− 1.276 to − 0.573		< 0.001***	< 0.001***	− 0.162
	Time: PMQ	− 0.023	− 0.351 to 0.304		0.889	1.000	− 0.004
Attention problems	Time	0.272	0.142 to 0.402	0.033	< 0.001***	< 0.001***	0.049
	PMQ	− 1.361	− 1.699 to − 1.023		< 0.001***	< 0.001***	− 0.247
	Time: PMQ	− 0.208	− 0.485 to 0.069		0.140	1.000	− 0.038
Rule-breaking behavior	Time	− 0.008	− 0.106 to 0.090	0.034	0.877	1.000	− 0.002
	PMQ	− 0.843	− 1.051 to − 0.636		< 0.001***	< 0.001***	− 0.219
	Time: PMQ	− 0.264	− 0.473 to − 0.056		0.013*	0.143	− 0.069
Aggressive behavior	Time	0.117	− 0.000 to 0.233	0.027	0.050	0.553	0.024
	PMQ	− 0.941	− 1.221 to − 0.661		< 0.001***	< 0.001***	− 0.196
	Time: PMQ	− 0.309	− 0.558 to − 0.060		0.015*	0.167	− 0.064
Anxious/depressed	Time	0.100	− 0.061 to 0.261	0.020	0.223	1.000	0.017
	PMQ	− 0.585	− 0.949 to − 0.222		0.002**	0.018*	− 0.099
	Time: PMQ	− 0.261	− 0.604 to 0.081		0.135	1.000	− 0.044

***p < 0.001, **p < 0.01, *p < 0.05.

**Figure 2:**
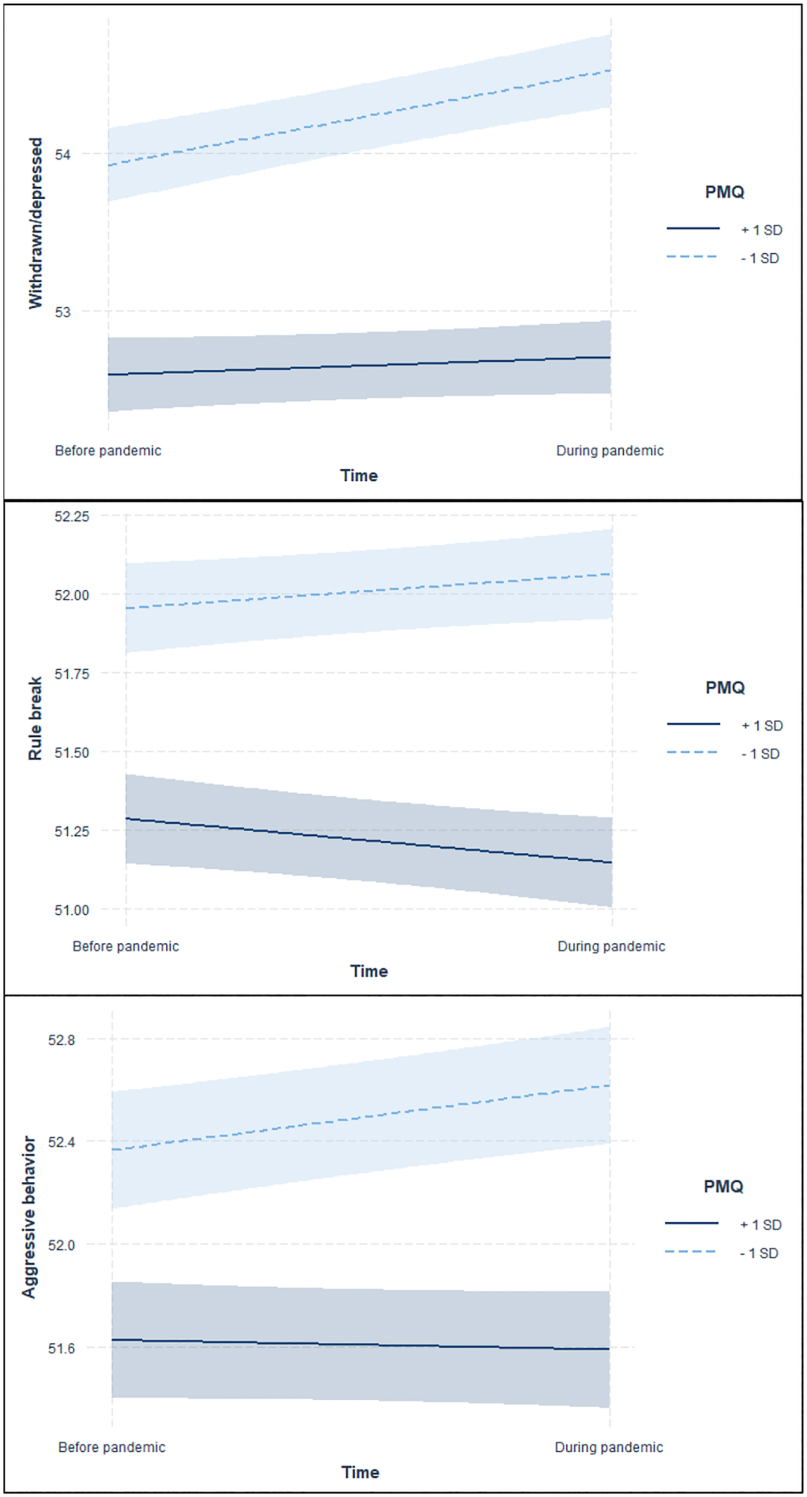
Simple slope analysis. Simple slopes analysis of cross-level interaction of PMQ and time point in mental health such as withdrawn/depressed, rule-breaking behavior, and aggressive behavior. Between the withdrawn/depressed and the time point, and aggressive behavior and the time point showed a significant positive association when PMQ was 1 SD below the mean. As well, the rule-breaking behavior and the time point showed a negative association when PMQ was 1 SD above the mean. *Notes.* SD: standard deviation; −1SD: 1SD below the mean of PMQ; +1SD: 1SD above the mean of PMQ; PMQ: Parental Monitoring Questionnaire.

The withdrawn/depressed and aggressive behavior of the CBCL showed a significant positive association with time point (i.e., worsened) when parent monitoring behavior was 1 SD below the mean, but there was no significant effect when parent monitoring behavior was 1 SD above the mean (see Table 4 and Fig. 2). Additionally, the rule-breaking behavior of the CBCL showed a significant negative association with time point (i.e., reduced) when parent monitoring behavior was 1 SD above the mean, but the change was not significant when it was 1 SD below the mean.

**Table 4:** Relationship between parent monitoring behavior strength and time point.

Outcome	Variable	B	95%CI	*R*^2^	*p* value
**CBCL**					
Withdrawn/depressed	1 SD above the mean of PMQ: Time	0.113	− 0.122 to 0.349	0.040	0.344
	1 SD below the mean of PMQ: Time	0.626	0.376 to 0.876		< 0.001***
Rule-breaking behavior	1 SD above the mean of PMQ: Time	− 0.138	− 0.276 to − 0.001	0.034	0.049 *
	1 SD below the mean of PMQ: Time	0.123	− 0.024 to 0.270		0.100
Aggressive behavior	1 SD above the mean of PMQ: Time	− 0.036	− 0.200 to 0.128	0.027	0.667
	1 SD below the mean of PMQ: Time	0.269	0.094 to 0.444		0.003***

*SD* standard deviation, *CBCL* child behavior checklist, *PMQ* parental monitoring questionnaire.

Furthermore, the group that exceeded the pre-pandemic CBCL reference value and the group that did not exceed it were analyzed separately (see Table S12 and Table S13 in the supplementary file) to investigate whether there was a difference in the change depending on the severity of the pre-pandemic CBCL. When CBCL scores before the pandemic exceeded the reference value, the main effects of time were significant (i.e., reduced) after multiple testing corrections at *p* < 0.001. In addition, in all scales after multiple testing corrections, no significant difference was observed for the main effects of PMQ and the interactions between parent monitoring behavior and the time point of the CBCL.

On the other hand, when the CBCL score before the pandemic did not exceed the reference value, the main effects of time were significant (i.e., worsened) after multiple testing corrections, excluding rule-breaking behavior. This finding includes somatic complaints (*p* < 0.01) and all others (i.e., total score, internalizing problem, externalizing problem, withdrawn/depressed, somatic complaints, social problems, thought problems, attention problems, aggressive behavior, anxious/depressed) (*p* < 0.001). Except for somatic complaints, the main effects of PMQ were significant after multiple testing corrections: anxious/depressed and social problems (*p* < 0.01), and all others (i.e., total score, internalizing problem, externalizing problem, withdrawn/depressed, thought problems, attention problems, and rule-breaking and aggressive behaviors) (*p* < 0.001). In addition, the interactions between parent monitoring behavior and the time of the withdrawn/depressed (*p* < 0.001), rule-breaking behaviors and aggressive behaviors (*p* < 0.01) were significant after multiple testing corrections.

In addition, the interactions between parent monitoring behavior (sub-item4: child disclosure) and time point of the withdrawn/depressed, rule-breaking behaviors were significant (*p* < 0.05). However, after multiple testing corrections, there were no significant differences (see Table S14). The result of simple slope analysis in PMQ sub-item 4 (child disclosure) was similar to that of the overall mean value of PMQ (see Table S15 and Fig. S2).

A significant difference was found in externalizing problem, social problems, attention problems, and rule-breaking behavior and aggressive behavior comparing the groups with and without third-year data (see Table S16).

## Discussion

The purpose of this study was to investigate the effect of the COVID-19 pandemic on the mental health of children and the effect of parental involvement during the pandemic. We used longitudinal observational data from a large sample of the ABCD Study. A total of 4885 children were analyzed in the study, adjusted for PMQ scores, and then analyzed for changes in CBCL scores before and after the onset of the pandemic. Our findings suggest that the COVID-19 pandemic has a minor adverse effect on children’s mental health. We also found that the degree of involvement between the child and the parent and parenting style affects a child’s mental health even in the face of the pandemic. For each, we will interpret the results obtained below.

First, we hypothesized that mental health problems in children would worsen over time due to the COVID-19 pandemic. The study results support our hypothesis. The emotional problem exacerbated by this pandemic was depression. This finding is consistent with previous studies and shows that depressive symptoms in children are exacerbated by the COVID-19 pandemic^30–35^, as is the case in adults^35–37^. However, the withdrawn/depressed behavior went from an average of 53.4 ± 5.7 pre-pandemic to 53.7 ± 5.9 since the onset of the pandemic, which is a change of only 0.3. Additionally, these values from before and during the COVID-19 pandemic are in the normal range. While the worsening of depressive tendencies for children was statistically significant, the magnitude of deterioration was clinically minor; hence, caution is warranted in the interpretation of these results. Next, this change was similar for attention, which worsened albeit only slightly clinically; the average attention problems went from 53.4 ± 5.4 pre-pandemic to 53.6 ± 5.6 since the onset of the pandemic. Interestingly, the results of our study did not uncover any effects on anxiety or physical complaints. Previous studies have reported that the COVID-19 pandemic does not exacerbate children’s emotional problems^38,39^. Rather, emotional problems of children aged 11–16 reportedly diminished in the United Kingdom^38^. A longitudinal study of about 1000 children in England reported a fair reduction in anxiety overall^39^. These results are consistent with our findings that the COVID-19 pandemic has no effect on children’s anxiety. Moreover, our findings showed that children’s behavioral problems were unaffected by the pandemic. This result is consistent with the findings of an online cross-sectional study of 1264 children (aged 2–6) and their parents in two primary schools in Hubei, China^40^. Hence, mental health problems, such as anxiety and behavioral issues commonly observed in children, seemed to be largely unaffected by the pandemic.

Additionally, we investigated whether there were differences in mental health changes between children who had already experienced severe symptoms prior to the pandemic and those who did not. Interestingly, if the mental health symptoms were initially severe, all mental health symptoms improved significantly during the pandemic. This improving trend might be a regression to the mean. Thus, it should be interpreted with caution. Nevertheless, this finding may indicate that children who initially had interpersonal problems moved away from the community, reducing their interpersonal problems and improving mental health. The previous study reported that children perceive home isolation positively rather than negatively, which reduces psychological distress and increases life satisfaction^41^. In contrast, children who did not exceed the cutoff before the pandemic had worsening mental health during the pandemic, excluding rule-breaking behavior. The interaction between lifestyle changes and the psychosocial stress of staying at home may further exacerbate the adverse effects on children's mental health^39^. However, the change in the *t*-score of CBCL was less than 1 point at maximum. Considering that the *t*-score is in the normal range, the pandemic may have hardly affected children whose mental health was initially stable. Most children have functioned at the same or higher levels since the pandemic and are satisfied with their current living conditions^41^.

Subsequently, we verified the hypothesis that parental involvement serves as a protective factor for a child’s mental health even during the pandemic. Parental involvement positively affected children’s mental health, emotional, and behavioral aspects. In particular, when parents and their children engaged in frequent conversations and parental understanding of their child’s condition was high, rule-breaking decreased; when such involvement was weak, the child’s depression and aggressive behavior increased. A cross-sectional study of 1655 parents and children in China found that parental attitudes and intimacy with children are positively correlated with the child’s mental or behavioral health^21^. These results are consistent with those of our study, which shows that parental child-rearing styles have a crucial impact on children’s mental health. Our study is a large longitudinal study of children living in the United States, which has the highest number of COVID-19 infections^42^. As far as we know, it is the first time that parental involvement has been shown to influence the mental health of children in the United States. During home confinement, children generally interact the most with parents and caregivers; so early detection and care of children’s mental health problems can prevent deterioration^43^. This finding may demonstrate that parents and caregivers impact their children’s mental health.

In particular, on analyzing each PMQ sub-item, it is found that less rule-breaking occurs when children communicate clearly with parents about their plans for school or activities with friends. In contrast, less disclosure is associated with increased depression. In other words, it might be necessary for parents and children to communicate frequently and fully about their daily lives, not merely about the negative aspects. Frequent conversations about the pandemic and expressions of unpleasant emotions affect externalizing and internalizing symptoms in children^44^. Rumination was positively associated with depressive symptoms in children aged 9 and 12^45^. Those who were not optimistic about the onset of a pandemic were at higher risk of depressive symptoms than those who were^46^. Therefore, encouraging parents to communicate more fully with their children and consider the positive aspects may prevent deteriorating mental health during the pandemic.

While our findings bring great benefits to this area of study, our research has limitations. First, we set March 1, 2020, as the start date of the COVID-19 pandemic, and analyzed those subjects who consented to provide third-year follow-up data from March 1, 2020, onwards. However, the period from March 1, 2020, to the date of actual data acquisition varies by subject, that is, the impact of the duration of the pandemic at the point of data collection has not been considered. In addition, whether there were various restrictions such as lockdowns and social interactions during these periods may vary depending on the area in which they live. The impact of the pandemic on children’s mental health may differ between the early stages of the pandemic and the stages of progress and recovery. In the future, it may be necessary to consider accumulative data across time included the mechanism. Second, we examined whether there was a difference between those participants with and without third-year data. We found that those without third-year data had slightly higher CBCL scores. Therefore, if the analysis had included people who withdrew from the study, overall child mental health may have been somewhat improved. Third, children’s mental health was investigated using the parent's assessment, and the degree of parental involvement was investigated using the children’s assessment in this study. In the future, both child and parental reports should be included to ensure data accuracy.

In conclusion, the results of this study indicate that the COVID-19 pandemic may slightly exacerbate depression and attention problems in children. On the other hand, if the mental health symptoms were severe before the pandemic, all mental health symptoms improved during the crisis. Additionally, even during the global public health crisis caused by COVID-19, positive parent–child relationships have a protective impact on pubescent children’s mental health in the United States. Therefore, increasing parent–child involvement is critical to children’s overall mental health even during the COVID-19 pandemic.

## Supplementary Information

Supplementary Information.

## Data Availability

The ABCD Study anonymized data, including all assessment domains, are released annually to the research community. Information on how to access ABCD data through the NDA is available on the ABCD Study data-sharing webpage: https://abcdstudy.org/scientists_data_sharing.html. Instructions on how to create an NDA study are available at https://nda.nih.gov/training/modules/study.html. The ABCD data repository grows and changes over time. The ABCD data used in this report came from the ABCD 4.0 data release (http://dx.doi.org/10.15154/1523041). R codes for the analyses can be accessed by (https://osf.io/jkeh6/?view_only=14151fb0609d478eadc932664d94c3c0).

## References

[1] Zhu N (2020). A novel coronavirus from patients with pneumonia in China, 2019. N. Engl. J. Med..

[2] World Health Organization (accessed 14 December 2021) https://covid19.who.int/.

[3] American Academy of Pediatrics. *Children and COVID-19:State data report*. Updated September 12, 2021 (accessed 9 December 2021) https://downloads.aap.org/AAP/PDF/AAP%20and%20CHA%20-%20Children%20and%20COVID-19%20State%20Data%20Report%2012.9%20FINAL.pdf.

[4] Lee J (2020). Mental health effects of school closures during COVID-19. Lancet Child Adolesc. Health.

[5] Xiang YT (2020). Timely mental health care for the 2019 novel coronavirus outbreak is urgently needed. Lancet Psychiatry.

[6] Leeb RT (2020). Mental health-related emergency department visits among children aged <18 years during the COVID-19 pandemic: United States, January 1–October 17, 2020. MMWR.

[7] Yard E (2021). Emergency Department visits for suspected suicide attempts among persons aged 12–25 years before and during the COVID-19 pandemic—United States, January 2019-May. MMWR.

[8] Racine N, McArthur BA, Cooke JE, Eirich R, Zhu J, Madigan S (2021). Global prevalence of depressive and anxiety symptoms in children and adolescents during COVID-19: A meta-analysis. JAMA Pediatr..

[9] Zhang L, Zhang D, Fang J, Wan Y, Tao F, Sun Y (2020). Assessment of mental health of Chinese Primary School Students before and after School Closing and Opening during the COVID-19 pandemic. JAMA Netw. Open..

[10] Vizard T (2020). Mental Health of Children and Young People in England 2020, Wave 1 Follow-Up to the 2017 Survey.

[11] Thorisdottir IE (2021). Depressive symptoms, mental wellbeing, and substance use among adolescents before and during the COVID-19 pandemic in Iceland: A longitudinal, population-based study. Lancet Psychiatry.

[12] Widnall, E. *et al*. *Young People’s mental health during the COVID-19 Pandemic 2020 *(accessed 26 December 2021) https://research-information.bris.ac.uk/ws/portalfiles/portal/248631996/Covid_Analysis_Report_24_08_2020.pdf.

[13] Bignardi G (2021). Longitudinal increases in childhood depression symptoms during the COVID-19 lockdown. Arch. Dis. Child..

[14] Davids EL, Roman NV, Leach L (2017). The link between parenting approaches and health behavior: A systematic review. J. Hum. Behav. Soc. Environ..

[15] Rohner RP, Britner PA (2002). Worldwide mental health correlates of parental acceptance–rejection: Review of cross-cultural and intracultural evidence. Cross-Cult. Res. J. Comp. Soc. Sci..

[16] Rohner RP, Khaleque A, Cournoyer DE (2005). Parental acceptance-rejection: Theory, methods, cross-cultural evidence, and implications. Ethos.

[17] Perrin EC, Leslie LK, Boat T (2016). Parenting as primary prevention. JAMA Pediatr..

[18] Anastopoulos AD, Shelton T, DuPaul GJ, Guevremont DC (1993). Parent training for attention deficit hyperactivity disorder: Its impact on parent functioning. J. Abnorm. Child Psychol..

[19] Serketich WJ, Dumas JE (1996). The effectiveness of behavioral parent training to modify antisocial behavior in children: A meta-analysis. Behav. Ther..

[20] Cobham VE, McDermott B, Haslam D, Sanders MR (2016). The role of parents, parenting and the family environment in children’s post-disaster mental health. Curr. Psychiatry Rep..

[21] Du F (2021). Associations between parent–child relationship, and children’s externalizing and internalizing symptoms, and lifestyle behaviors in China during the COVID-19 epidemic. Sci Rep..

[22] Jernigan TL, Brown SA (2018). ABCD consortium coordinators, ABCD consortium coordinators [introduction]. Introduction. Dev. Cogn. Neurosci..

[23] Garavan H (2018). Recruiting the ABCD sample: Design considerations and procedures. Dev. Cogn. Neurosci..

[24] Paul SE (2021). Associations between prenatal cannabis exposure and childhood outcomes: Results from the ABCD study. JAMA Psychiatry.

[25] Achenbach TM (2009). The Achenbach System of Empirically Based Assessment (ASEBA): Development, Findings, Theory and Applications.

[26] Achenbach, T. M., Rescorla, L. A., University of Vermont, Research Center for Children Youth & Families. Manual for the ASEBA school-age forms and profiles: Child Behavior Checklist for ages: 6–18. *Teacher’s Report Form, Youth Self-Report: An Integrated System of Multi-Informant Assessment*. ASEBA (2001).

[27] Zucker RA (2018). Assessment of culture and environment in the Adolescent Brain and Cognitive Development Study: Rationale, description of measures, and early data. Dev. Cogn. Neurosci..

[28] R Development Core Team. R Foundation for Statistical Computing, Vienna, Austria (2020).

[29] Kuznetsova A, Brockhoff PB, Christensen RHB (2017). lmerTest package: Tests in linear mixed effects models. J. Stat. Soft..

[30] Xie X (2020). Mental health status among children in home confinement during the Coronavirus disease 2019 outbreak in Hubei Province, China. JAMA Pediatr..

[31] Chen F (2020). Depression and anxiety among adolescents during COVID-19: A cross-sectional study. Brain Behav. Immun..

[32] Zhou SJ (2020). Prevalence and socio-demographic correlates of psychological health problems in Chinese adolescents during the outbreak of COVID-19. Eur. Child Adolesc. Psychiatry.

[33] Liu X (2020). Psychological status and behavior changes of the public during the COVID-19 epidemic in China. Infect. Dis. Poverty.

[34] Duan L (2020). An investigation of mental health status of children and adolescents in China during the outbreak of COVID-19. J. Affect. Disord..

[35] COVID-19 Mental Disorders Collaborators (2021). Global prevalence and burden of depressive and anxiety disorders in 204 countries and territories in 2020 due to the COVID-19 pandemic. Lancet.

[36] Salari N (2020). Prevalence of stress, anxiety, depression among the general population during the COVID-19 pandemic: A systematic review and meta-analysis. Glob. Health.

[37] Matsumoto K, Hamatani S, Shimizu E, Käll A, Andersson G (2022). Impact of post-COVID conditions on mental health: A cross-sectional study in Japan and Sweden. BMC Psychiatry.

[38] Waite PD, Pearcey S, Shum A, Raw J, Patalay P, Creswell C (2020). How did the mental health of children and adolescents change during early lockdown during the COVID-19 pandemic in the UK?. Psychiatry.

[39] Wang G, Zhang Y, Zhao J, Zhang J, Jiang F (2020). Mitigate the effects of home confinement on children during the COVID-19 outbreak. Lancet.

[40] Liu Q (2021). The prevalence of behavioral problems among school-aged children in home quarantine during the COVID-19 pandemic in China. J. Affect. Disord..

[41] Tang S, Xiang M, Cheung T, Xiang YT (2021). Mental health and its correlates among children and adolescents during COVID-19 school closure: The importance of parent-child discussion. J. Affect. Disord..

[42] Worldmeter (accessed 3 November 2022) https://www.worldometers.info/coronavirus/countries-where-coronavirus-has-spread/.

[43] Zwi M, Jones H, Thorgaard C, York A, Dennis JA (2011). Parent training interventions for Attention Deficit Hyperactivity Disorder (ADHD) in children aged 5 to 18 years. Cochrane Database Syst. Rev..

[44] Du F, He L, Francis MR (2021). Associations between parent–child relationship, and children’s externalizing and internalizing symptoms, and lifestyle behaviors in China during the COVID-19 epidemic. Sci. Rep..

[45] Lionetti F, Klein DN, Pastore M, Aron EN, Aron A, Pluess M (2021). The role of environmental sensitivity in the development of rumination and depressive symptoms in childhood: A longitudinal study. Eur. Child Adolesc. Psychiatry..

[46] Xie X, Xue Q, Zhou Y, Zhu K, Liu Q, Zhang J, Song R (2020). Mental health status among children in home confinement during the coronavirus disease 2019 outbreak in Hubei Province, China. JAMA Pediatr..

