# Supplementary material for: Longitudinal impact of COVID-19 pandemic on mental health of children in the ABCD study cohort
Source: Sci Rep. 2022 Nov 15;12:19601. doi: 10.1038/s41598-022-22694-z (PMC9665012; doi:10.1038/s41598-022-22694-z)
Supplement: Supplementary file 1 — Supplementary Information. [file 41598_2022_22694_MOESM1_ESM.docx]

**Supplementary Materials**

**Figure S1.** **Flow-chart of the sampling procedure for the ABCD Study population used in this study (*n*=4702)**

**
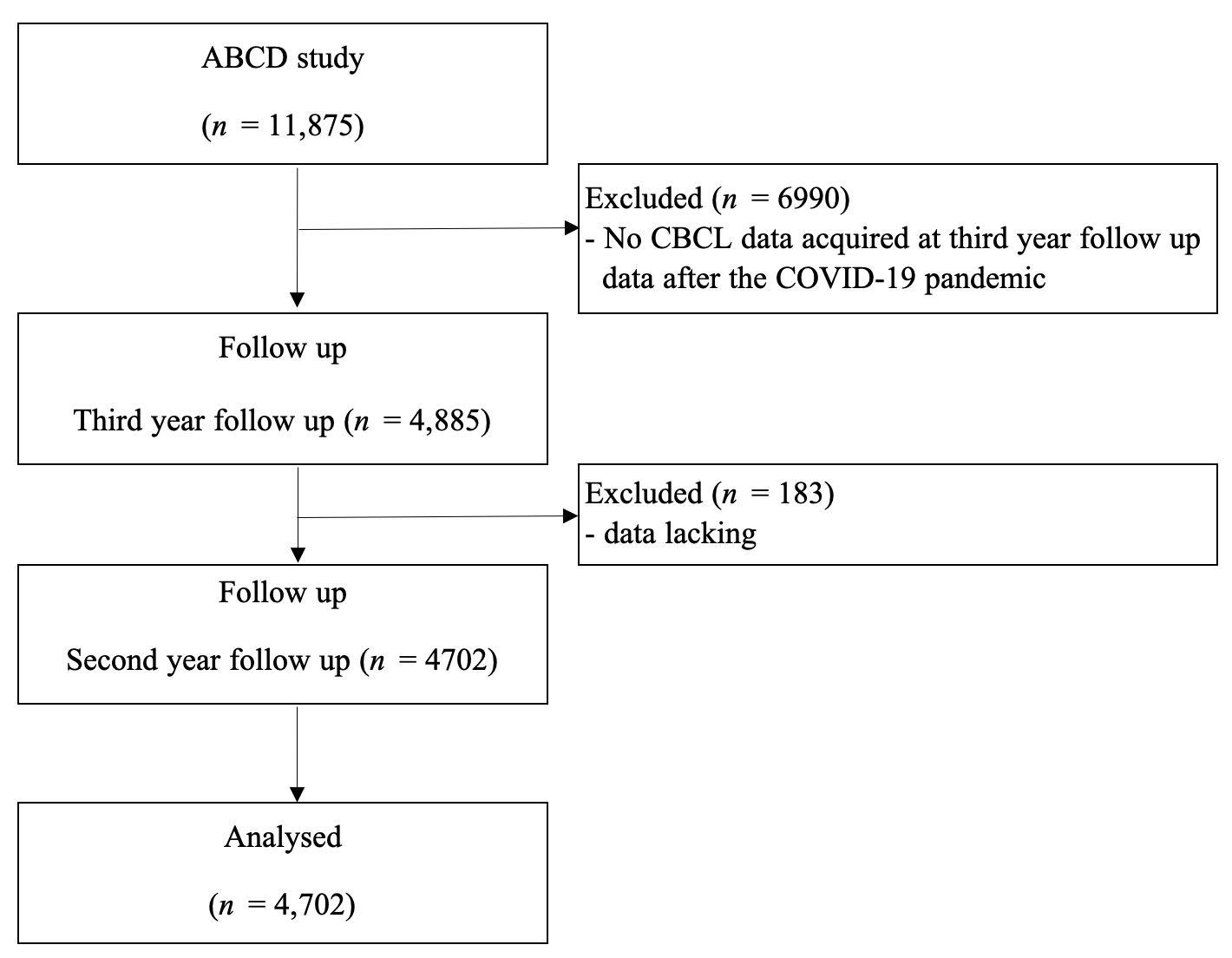
**

| **Table S1.** **Full Regression Results: Effects of Parent Monitoring and Time Point on Total score of CBCL** | | | | | |
| --- | --- | --- | --- | --- | --- |
| Fixed effects | B | 95%CI | *R^2^* | *p* | *β* |
| Time | 0.208 | -0.043 to 0.459 | 0.050 | 0.105 | 0.019 |
| PMQ | -2.636 | -3.297 to -1.975 |  | 0.000 | -0.237 |
| White | -0.534 | -1.725 to 0.656 |  | 0.379 | -0.024 |
| Black | -4.431 | -6.196 to -2.667 |  | 0.000 | -0.122 |
| Hispanic | -1.552 | -3.002 to -0.102 |  | 0.036 | -0.055 |
| Asian | -6.425 | -8.968 to -3.882 |  | 0.000 | -0.088 |
| Sex | -0.167 | -0.811 to 0.476 |  | 0.610 | -0.008 |
| Income | -0.554 | -0.905 to -0.203 |  | 0.002 | -0.07 |
| Education_mother | 0.115 | -0.073 to 0.302 |  | 0.230 | 0.026 |
| Education_father | -0.114 | -0.276 to 0.048 |  | 0.169 | -0.029 |
| Twin | -3.528 | -4.629 to -2.427 |  | 0.000 | -0.126 |
| Time:PMQ | -0.213 | -0.749 to 0.322 |  | 0.435 | -0.019 |
| Random intercepts | Std. Dev. |  |  |  |  |
| Individual | 4.147 |  |  |  |  |
| Sibling | 8.028 |  |  |  |  |
| Location | 0.939 |  |  |  |  |

| **Table S2**. **Full Regression Results: Effects of Parent Monitoring and Time Point on Internalizing Problem of CBCL** | | | | | |
| --- | --- | --- | --- | --- | --- |
| Fixed effects | B | 95%CI | *R^2^* | *p* | *β* |
| Time | 0.139 | -0.132 to 0.411 | 0.040 | 0.315 | 0.013 |
| PMQ | -1.872 | -2.510 to -1.234 |  | 0.000 | -0.179 |
| White | -0.662 | -1.775 to 0.450 |  | 0.243 | -0.031 |
| Black | -5.360 | -7.004 to -3.715 |  | 0.000 | -0.157 |
| Hispanic | -1.448 | -2.796 to -0.100 |  | 0.035 | -0.055 |
| Asian | -5.005 | -7.399 to -2.610 |  | 0.000 | -0.073 |
| Sex | 0.441 | -0.174 to 1.056 |  | 0.016 | 0.021 |
| Income | -0.610 | -0.937 to -0.283 |  | 0.000 | -0.082 |
| Education_mother | 0.149 | 0.027 to -0.324 |  | 0.097 | 0.036 |
| Education_father | -0.005 | -0.157 to -0.147 |  | 0.953 | -0.001 |
| Twin | -2.974 | -3.956 to -1.993 |  | 0.000 | -0.113 |
| Time:PMQ | -0.176 | -0.755 to 0.404 |  | 0.553 | -0.017 |
| Random intercepts | Std. Dev. |  |  |  |  |
| Individual | 4.393 |  |  |  |  |
| Sibling | 6.981 |  |  |  |  |
| Location | 0.646 |  |  |  |  |

| **Table S3.** **Full Regression Results: Effects of Parent Monitoring and Time Point on Externalizing problem of CBCL** | | | | | |
| --- | --- | --- | --- | --- | --- |
| Fixed effects | B | 95%CI | *R^2^* | *p* | *β* |
| Time | 0.251 | 0.020 to 0.481 | 0.036 | 0.033 | 0.026 |
| PMQ | -2.206 | -2.785 to -1.627 |  | 0.000 | -0.231 |
| White | -0.302 | -1.302 to 0.698 |  | 0.553 | -0.016 |
| Black | -1.766 | -3.245 to -0.287 |  | 0.019 | -0.057 |
| Hispanic | -0.640 | -1.861 to 0.580 |  | 0.304 | -0.026 |
| Asian | -3.064 | -5.223 to -0.906 |  | 0.005 | -0.049 |
| Sex | -0.762 | -1.318 to -0.207 |  | 0.007 | 0.040 |
| Income | -0.356 | - 0.650 to -0.062 |  | 0.018 | -0.052 |
| Education_mother | 0.023 | -0.135 to 0.181 |  | 0.776 | 0.006 |
| Education_father | -0.126 | -0.263 to 0.010 |  | 0.069 | -0.037 |
| Twin | -2.081 | -3.001 to -1.160 |  | 0.000 | -0.086 |
| Time:PMQ | -0.396 | -0.887 to 0.095 |  | 0.114 | -0.041 |
| Random intercepts | Std. Dev. |  |  |  |  |
| Individual | 4.593 |  |  |  |  |
| Sibling | 5.940 |  |  |  |  |
| Location | 0.860 |  |  |  |  |

| **Table S4**. **Full Regression Results: Effects of Parent Monitoring and Time Point on Withdrawn Depressed of CBCL** | | | | | |
| --- | --- | --- | --- | --- | --- |
| Fixed effects | B | 95%CI | *R^2^* | *p* | *β* |
| Time | 0.370 | 0.203 to 0.537 | 0.040 | 0.000 | 0.064 |
| PMQ | -1.669 | -2.021 to -1.317 |  | 0.000 | -0.290 |
| White | -0.657 | -1.247 to -0.068 |  | 0.029 | -0.056 |
| Black | -1.906 | -2.774 to -1.038 |  | 0.000 | -0.102 |
| Hispanic | -0.602 | -1.311 to 0.106 |  | 0.096 | -0.041 |
| Asian | -1.689 | -2.973 to -0.405 |  | 0.010 | -0.045 |
| Sex | 0.072 | -0.263 to 0.407 |  | 0.674 | 0.006 |
| Income | -0.303 | -0.476 to -0.131 |  | 0.001 | -0.074 |
| Education_mother | 0.016 | -0.077 to 0.109 |  | 0.731 | 0.007 |
| Education_father | -0.019 | -0.100 to 0.062 |  | 0.644 | -0.009 |
| Twin | -1.111 | -1.584 to -0.638 |  | 0.000 | -0.077 |
| Time:PMQ | -0.518 | -0.874 to -0.162 |  | 0.004 | -0.090 |
| Random intercepts | Std. Dev. |  |  |  |  |
| Individual | 2.914 |  |  |  |  |
| Sibling | 3.111 |  |  |  |  |
| Location | 0.167 |  |  |  |  |

| **Table S5.** **Full Regression Results: Effects of Parent Monitoring and Time Point on Somatic Complaints of CBCL** | | | | | |
| --- | --- | --- | --- | --- | --- |
| Fixed effects | B | 95%CI | *R^2^* | *p* | *β* |
| Time | -0.132 | -0.309 to 0.044 | 0.029 | 0.142 | -0.023 |
| PMQ | -0.346 | -0.682 to -0.010 |  | 0.044 | -0.060 |
| White | -0.506 | -1.089 to 0.076 |  | 0.088 | -0.043 |
| Black | -2.399 | -3.259 to -1.539 |  | 0.000 | -0.128 |
| Hispanic | -0.794 | -1.500 to -0.089 |  | 0.027 | -0.054 |
| Asian | -2.302 | -3.560 to -1.045 |  | 0.000 | -0.061 |
| Sex | 0.450 | 0.127 to 0.773 |  | 0.006 | 0.039 |
| Income | -0.325 | -0.496 to -0.154 |  | 0.000 | -0.079 |
| Education_mother | 0.010 | -0.082 to 0.102 |  | 0.834 | 0.004 |
| Education_father | -0.051 | -0.130 to 0.029 |  | 0.212 | -0.025 |
| Twin | -1.406 | -1.917 to -0.896 |  | 0.000 | -0.097 |
| Time:PMQ | -0.088 | -0.464 to 0.289 |  | 0.648 | -0.015 |
| Random intercepts | Std. Dev. |  |  |  |  |
| Individual | 1.811 |  |  |  |  |
| Sibling | 3.581 |  |  |  |  |
| Location | 0.327 |  |  |  |  |

| **Table S6.** **Full Regression Results: Effects of Parent Monitoring and Time Point on Social Problems of CBCL** | | | | | |
| --- | --- | --- | --- | --- | --- |
| Fixed effects | B | 95%CI | *R^2^* | *p* | *β* |
| Time | -0.002 | -0.128 to 0.124 | 0.023 | 0.976 | 0.000 |
| PMQ | -0.611 | -0.893 to -0.329 |  | 0.000 | -0.129 |
| White | 0.008 | -0.469 to 0.485 |  | 0.974 | 0.001 |
| Black | -0.636 | -1.339 to 0.066 |  | 0.076 | -0.041 |
| Hispanic | -0.556 | -1.131 to 0.019 |  | 0.058 | -0.046 |
| Asian | -1.299 | -2.334 to -0.264 |  | 0.014 | -0.042 |
| Sex | -0.233 | -0.502 to 0.036 |  | 0.090 | -0.024 |
| Income | -0.336 | -0.476 to -0.196 |  | 0.000 | -0.099 |
| Education_mother | 0.006 | -0.069 to 0.081 |  | 0.871 | 0.003 |
| Education_father | -0.039 | -0.104 to 0.027 |  | 0.246 | -0.023 |
| Twin | -0.639 | -1.036 to -0.241 |  | 0.002 | -0.053 |
| Time:PMQ | 0.043 | -0.226 to 0.311 |  | 0.754 | 0.009 |
| Random intercepts | Std. Dev. |  |  |  |  |
| Individual | 2.316 |  |  |  |  |
| Sibling | 2.631 |  |  |  |  |
| Location | 0.199 |  |  |  |  |

| **Table S7.** **Full Regression Results: Effects of Parent Monitoring and Time Point on Thought Problems of CBCL** | | | | | |
| --- | --- | --- | --- | --- | --- |
| Fixed effects | B | 95%CI | *R^2^* | *p* | *β* |
| Time | -0.012 | -0.166 to 0.141 | 0.027 | 0.875 | -0.002 |
| PMQ | -0.925 | -1.276 to -0.573 |  | 0.000 | -0.162 |
| White | -0.319 | -0.916 to 0.279 |  | 0.296 | -0.028 |
| Black | -2.119 | -3.001 to -1.238 |  | 0.000 | -0.114 |
| Hispanic | -1.104 | -1.830 to -0.377 |  | 0.003 | -0.076 |
| Asian | -1.803 | -3.098 to -0.507 |  | 0.006 | -0.048 |
| Sex | -0.063 | -0.398 to 0.272 |  | 0.713 | -0.006 |
| Income | -0.314 | -0.490 to -0.139 |  | 0.000 | -0.078 |
| Education_mother | 0.047 | -0.047 to 0.142 |  | 0.326 | 0.021 |
| Education_father | -0.001 | -0.083 to 0.080 |  | 0.976 | -0.001 |
| Twin | -1.256 | -1.784 to -0.728 |  | 0.000 | -0.087 |
| Time:PMQ | -0.023 | -0.351 to 0.304 |  | 0.889 | -0.004 |
| Random intercepts | Std. Dev. |  |  |  |  |
| Individual | 2.896 |  |  |  |  |
| Sibling | 3.311 |  |  |  |  |
| Location | 0.404 |  |  |  |  |

**Table S8.** **Full Regression Results: Effects of Parent Monitoring and Time Point on Attention Problems of CBCL**

| Fixed effects | B | 95%CI | *R^2^* | *p* | *β* |
| --- | --- | --- | --- | --- | --- |
| Time | 0.272 | 0.142 to 0.402 | 0.033 | 0.000 | 0.049 |
| PMQ | -1.361 | -1.699 to -1.023 |  | 0.000 | -0.247 |
| White | -0.448 | -1.027 to 0.132 |  | 0.130 | -0.040 |
| Black | -1.233 | -2.088 to -0.377 |  | 0.005 | -0.069 |
| Hispanic | -1.035 | -1.734 to -0.336 |  | 0.004 | -0.074 |
| Asian | -2.084 | -3.336 to -0.833 |  | 0.001 | -0.058 |
| Sex | -0.031 | -0.355 to 0.294 |  | 0.853 | -0.003 |
| Income | -0.229 | -0.399 to -0.059 |  | 0.008 | -0.058 |
| Education_mother | 0.030 | -0.062 to 0.121 |  | 0.527 | 0.014 |
| Education_father | -0.079 | -0.158 to 0.000 |  | 0.051 | -0.040 |
| Twin | -1.032 | -1.523 to -0.540 |  | 0.000 | -0.074 |
| Time:PMQ | -0.208 | -0.485 to 0.069 |  | 0.140 | -0.038 |
| Random intercepts | Std. Dev. |  |  |  |  |
| Individual | 2.757 |  |  |  |  |
| Sibling | 3.446 |  |  |  |  |
| Location | 0.255 |  |  |  |  |

| **Table S9.** **Full Regression Results: Effects of Parent Monitoring and Time Point on Rule-Breaking Behavior of CBCL** | | | | | |
| --- | --- | --- | --- | --- | --- |
| Fixed effects | B | 95%CI | *R^2^* | *p* | *β* |
| Time | -0.008 | -0.106 to 0.090 | 0.034 | 0.877 | -0.002 |
| PMQ | -0.843 | -1.051 to -0.636 |  | 0.000 | -0.219 |
| White | -0.437 | 0.794 to -0.080 |  | 0.016 | -0.056 |
| Black | -0.331 | -0.857 to 0.196 |  | 0.218 | -0.026 |
| Hispanic | -0.674 | -1.104 to -0.245 |  | 0.002 | -0.069 |
| Asian | -0.948 | -1.718 to -0.177 |  | 0.016 | -0.037 |
| Sex | 0.023 | -0.176 to 0.222 |  | 0.820 | 0.003 |
| Income | -0.153 | -0.258 to -0.049 |  | 0.004 | -0.056 |
| Education_mother | -0.029 | -0.085 to 0.027 |  | 0.315 | -0.019 |
| Education_father | -0.045 | -0.094 to 0.004 |  | 0.072 | -0.033 |
| Twin | -0.509 | -0.808 to -0.210 |  | 0.001 | -0.052 |
| Time:PMQ | -0.264 | -0.473 to -0.056 |  | 0.013 | -0.069 |
| Random intercepts | Std. Dev. |  |  |  |  |
| Individual | 1.390 |  |  |  |  |
| Sibling | 2.169 |  |  |  |  |
| Location | 0.133 |  |  |  |  |

| **Table S10.** **Full Regression Results: Effects of Parent Monitoring and Time Point on Aggressive Behavior of CBCL** | | | | | |
| --- | --- | --- | --- | --- | --- |
| Fixed effects | B | 95%CI | *R^2^* | *p* | *β* |
| Time | 0.117 | -0.000 to 0.233 | 0.027 | 0.050 | 0.024 |
| PMQ | -0.941 | -1.221 to -0.661 |  | 0.000 | -0.196 |
| White | -0.115 | -0.595 to 0.366 |  | 0.640 | -0.012 |
| Black | -0.676 | -1.386 to 0.034 |  | 0,062 | -0.043 |
| Hispanic | -0.347 | -0.933 to 0.239 |  | 0.246 | -0.029 |
| Asian | -0.809 | -1.848 to 0.229 |  | 0.127 | -0.026 |
| Sex | -0.436 | -0.704 to -0.168 |  | 0.001 | -0.045 |
| Income | -0.205 | -0.346 to -0.064 |  | 0.005 | -0.060 |
| Education_mother | 0.031 | -0.044 to 0.107 |  | 0.418 | 0.017 |
| Education_father | -0.052 | -0.118 to 0.013 |  | 0.117 | -0.03 |
| Twin | -0.749 | -1.186 to -0.312 |  | 0.001 | -0.062 |
| Time:PMQ | -0.309 | -0.558 to -0.060 |  | 0.015 | -0.064 |
| Random intercepts | Std. Dev. |  |  |  |  |
| Individual | 2.245 |  |  |  |  |
| Sibling | 2.778 |  |  |  |  |
| Location | 0.386 |  |  |  |  |

| **Table S11. Full Regression Results: Effects of Parent Monitoring and Time Point on Anxious/depressed of CBCL** | | | | | |
| --- | --- | --- | --- | --- | --- |
| Fixed effects | B | 95%CI | *R^2^* | *p* | *β* |
| Time | 0.100 | -0.061 to 0.261 | 0.020 | 0.223 | 0.017 |
| PMQ | -0.585 | -0.949 to -0.222 |  | 0.002 | -0.099 |
| White | -0.115 | -0.729 to 0.499 |  | 0.714 | -0.010 |
| Black | -2.295 | -3.201 to -1.389 |  | 0.000 | -0.120 |
| Hispanic | -0.682 | -1.427 to 0.064 |  | 0.073 | -0.046 |
| Asian | -1.599 | -2.933 to -0.264 |  | 0.019 | -0.041 |
| Sex | 0.183 | -0.163 to 0.529 |  | 0.300 | 0.016 |
| Income | -0.357 | -0.538 to -0.177 |  | 0.000 | -0.085 |
| Education_mother | 0.073 | -0.024 to 0.170 |  | 0.139 | 0.031 |
| Education_father | 0.015 | -0.069 to 0.098 |  | 0.733 | 0.007 |
| Twin | -0.987 | -1.519 to -0.455 |  | 0.000 | -0.066 |
| Time:PMQ | -0.261 | -0.604 to 0.081 |  | 0.135 | -0.044 |
| Random intercepts | Std. Dev. |  |  |  |  |
| Individual | 3.082 |  |  |  |  |
| Sibling | 3.300 |  |  |  |  |
| Location | 0.369 |  |  |  |  |

**Table S12.** **Parental monitoring and timely impact on children’s mental health: CBCL score of group exceeds the reference value before the pandemic**

| Outcome | | Variable | | B | 95%CI | | *R^2^* | *p* value | Bonferroni corrected *p* value |
| --- | --- | --- | --- | --- | --- | --- | --- | --- | --- |
| CBCL | Total score | Time | | -3.883 | -4.689 to -3.078 | | 0.107 | < 0.001*** | < 0.001*** |
|  |  | PMQ | | -0.044 | -1.110 to 1.022 | |  | 0.936 | 1.000 |
|  |  | Time: PMQ | | -1.377 | -2.853 to 0.099 | |  | 0.068 | 0.748 |
|  | Internalizing problem | Time | | -4.697 | -5.421 to -3.973 | | 0.124 | < 0.001*** | < 0.001*** |
|  |  | PMQ | | -0.100 | -1.127 to 0.927 | |  | 0.849 | 1.000 |
|  |  | Time: PMQ | | -0.994 | -2.378 to 0.388 | |  | 0.159 | 1.000 |
|  | Externalizing problem | Time | | -5.505 | -6.581 to -4.429 | | 0.164 | < 0.001*** | < 0.001*** |
|  |  | PMQ | | -0.420 | -1.915 to 1.074 | |  | 0.582 | 1.000 |
|  |  | Time: PMQ | | -2.012 | -3.932 to -0.092 | |  | 0.041 | 0.451 |
|  | Withdrawn/depressed | Time | | -6.200 | -7.476 to -4.924 | | 0.165 | < 0.001*** | < 0.001*** |
|  |  | PMQ | | -1.350 | -3.076 to 0.376 | |  | 0.127 | 1.000 |
|  |  | Time: PMQ | | -1.644 | -4.172 to 0.884 | |  | 0.204 | 1.000 |
|  | Somatic complaints | Time | | -6.318 | -7.245 to -5.390 | | 0.234 | < 0.001*** | < 0.001*** |
|  |  | PMQ | | -0.029 | -1.312 to 1.370 | |  | 0.966 | 1.000 |
|  |  | Time: PMQ | | -1.757 | -3.634 to 0.121 | |  | 0.068 | 0.748 |
|  | Social problems | Time | | -5.974 | -7.183 to -4.765 | | 0.232 | < 0.001*** | < 0.001*** |
|  |  | PMQ | | 0.294 | -1.273 to 1.861 | |  | 0.714 | 1.000 |
|  |  | Time: PMQ | | 1.166 | -0.892 to 3.223 | |  | 0.269 | 1.000 |
|  | Thought problems | Time | | -5.879 | -6.815 to -4.943 | | 0.216 | < 0.001*** | < 0.001*** |
|  |  | PMQ | | -0.593 | -1.755 to 0.569 | |  | 0.319 | 1.000 |
|  |  | Time: PMQ | | -1.455 | -3.205 to 0.296 | |  | 0.105 | 1.000 |
|  | Attention problems | Time | | -4.602 | -5.781 to -3.424 | | 0.155 | < 0.001*** | < 0.001*** |
|  |  | PMQ | | -1.731 | -3.281 to -0.181 | |  | 0.030* | 0.331 |
|  |  | Time: PMQ | | -2.025 | -4.202 to 0.151 | |  | 0.070 | 0.769 |
|  | Rule-breaking behavior | Time | | -7.149 | -8.617 to -5.681 | | 0.468 | < 0.001*** | < 0.001*** |
|  |  | PMQ | | 1.049 | -1.149 to 3.247 | |  | 0.355 | 1.000 |
|  |  | Time: PMQ | | 0.642 | -2.059 to 3.344 | |  | 0.643 | 1.000 |
|  | Aggressive behavior | Time | | -5.907 | -7.446 to -4.368 | | 0.206 | < 0.001*** | < 0.001*** |
|  |  | PMQ | | 0.477 | -1.581 to 2.536 | |  | 0.651 | 1.000 |
|  |  | Time: PMQ | | -1.735 | -4.500 to 1.029 | |  | 0.221 | 1.000 |
|  | Anxious/depressed | Time | | -5.542 | - 6.633 to -4.450 | | 0.139 | < 0.001*** | < 0.001*** |
|  |  | PMQ | | 0.657 | -0.855 to 2.168 | |  | 0.395 | 1.000 |
|  |  | Time: PMQ | | -0.767 | -2.760 to 1.225 | |  | 0.451 | 1.000 |
| ****p*<0.001, ***p*<0.01, **p*<0.05 | | |  | | |  |  |  |  |

**Table S13.** **Parental monitoring and timely impact on children’s mental health: CBCL score of group did not exceed the reference value before the pandemic**

| Outcome | | Variable | B | 95%CI | *R^2^* | *p* value | Bonferroni corrected *p* value |
| --- | --- | --- | --- | --- | --- | --- | --- |
| CBCL | Total score | Time | 0.606 | 0.344 to 0.868 | 0.039 | < 0.001*** | < 0.001*** |
|  |  | PMQ | -2.126 | -2.738 to -1.514 |  | < 0.001*** | < 0.001*** |
|  |  | Time: PMQ | -0.397 | -0.965 to 0.172 |  | 0.172 | 1.000 |
|  | Internalizing problem | Time | 0.893 | 0.607 to 1.179 | 0.032 | < 0.001*** | < 0.001*** |
|  |  | PMQ | -1.331 | -1.903 to -0.760 |  | < 0.001*** | < 0.001*** |
|  |  | Time: PMQ | -0.497 | -1.119 to 0.125 |  | 0.117 | 1.000 |
|  | Externalizing problem | Time | 0.603 | 0.371 to 0.835 | 0.028 | < 0.001*** | < 0.001*** |
|  |  | PMQ | -1.703 | -2.235 to -1.171 |  | < 0.001*** | < 0.001*** |
|  |  | Time: PMQ | -0.534 | -1.036 to -0.033 |  | 0.037* | 0.407 |
|  | Withdrawn/depressed | Time | 0.795 | 0.644 to 0.946 | 0.043 | < 0.001*** | < 0.001*** |
|  |  | PMQ | -1.281 | -1.542 to -1.020 |  | < 0.001*** | < 0.001*** |
|  |  | Time: PMQ | -0.769 | -1.093 to -0.446 |  | < 0.001*** | < 0.001*** |
|  | Somatic complaints | Time | 0.305 | 0.137 to 0.474 | 0.020 | < 0.001*** | 0.004* |
|  |  | PMQ | -0.305 | -0.586 to -0.023 |  | 0.034 | 0.374 |
|  |  | Time: PMQ | -0.043 | -0.404 to 0.317 |  | 0.813 | 1.000 |
|  | Social problems | Time | 0.237 | 0.119 to 0.354 | 0.018 | < 0.001*** | < 0.001*** |
|  |  | PMQ | -0.445 | -0.669 to -0.221 |  | < 0.001*** | 0.001** |
|  |  | Time: PMQ | -0.236 | -0.488 to 0.016 |  | 0.067 | 0.737 |
|  | Thought problems | Time | 0.399 | 0.257 to 0.540 | 0.027 | < 0.001*** | < 0.001*** |
|  |  | PMQ | -0.575 | -0.839 to -0.311 |  | < 0.001*** | < 0.001*** |
|  |  | Time: PMQ | -0.153 | -0.458 to 0.152 |  | 0.325 | 1.000 |
|  | Attention problems | Time | 0.507 | 0.388 to 0.626 | 0.028 | < 0.001*** | < 0.001*** |
|  |  | PMQ | -1.046 | -1.312 to -0.781 |  | < 0.001*** | < 0.001*** |
|  |  | Time: PMQ | -0.289 | -0.544 to -0.033 |  | 0.271 | 1.000 |
|  | Rule-breaking behavior | Time | 0.103 | 0.010 to 0.197 | 0.031 | 0.030* | 0.330 |
|  |  | PMQ | -0.751 | -0.934 to -0.569 |  | < 0.001*** | < 0.001*** |
|  |  | Time: PMQ | -0.368 | -0.567 to -0.169 |  | < 0.001*** | 0.003** |
|  | Aggressive behavior | Time | 0.321 | 0.215 to 0.426 | 0.026 | < 0.001*** | < 0.001*** |
|  |  | PMQ | -0.735 | -0.947 to -0.523 |  | < 0.001*** | < 0.001*** |
|  |  | Time: PMQ | -0.400 | -0.626 to -0.174 |  | < 0.001*** | 0.006** |
|  | Anxious/depressed | Time | 0.512 | 0.367 to 0.658 | 0.020 | < 0.001*** | < 0.001*** |
|  |  | PMQ | -0.506 | -0.764 to -0.247 |  | <0.001*** | 0.001* |
|  |  | Time: PMQ | -0.370 | -0.683 to 0.057 |  | 0.021* | 0.231 |
| ****p*<0.001, ***p*<0.01, **p*<0.05 | | | | | | | |

**Table S14**. **Effects of Parent Monitoring by Sub-item and Time Point on Mental Health in Children**

| Outcome | | Variable | B | 95%CI | *R^2^* | *p* value | Bonferroni corrected *p* value |
| --- | --- | --- | --- | --- | --- | --- | --- |
| CBCL | Withdrawn/depressed | Time | 0.362 | 0.194 to 0.531 | 0.045 | < 0.001*** | < 0.001*** |
|  |  | PMQ 1  PMQ 2  PMQ 3  PMQ 4  PMQ 5 | -0.283  0.045  -0.219  -0.644  -0.309 | -0.641 to 0.075  -0.266 to 0.356  -0.542 to 0.104  -0.810 to -0.478  -0.483 to -0.135 |  | 0.121  0.776  0.185  < 0.001***  < 0.001*** | 1.000  1.000  1.000  < 0.001***  <0.01** |
|  |  | Time: PMQ 1  Time: PMQ 2  Time: PMQ 3  Time: PMQ 4  Time: PMQ 5 | 0.027  -0.238  -0.028  -0.182  -0.027 | -0.351 to 0.406  -0.565 to 0.090  -0.366 to 0.310  -0.353 to -0.010  -0.202 to 0.148 |  | 0.887  0.155  0.871  0.038*  0.765 | 1.000  1.000  1.000  0.418  1.000 |
|  | Rule-breaking behavior | Time | -0.017 | -0.115 to 0.082 | 0.034 | 0.739 | 1.000 |
|  |  | PMQ 1  PMQ 2  PMQ 3  PMQ 4  PMQ 5 | -0.222  -0.189  -0.155  -0.178  -0.117 | -0.432 to -0.013  -0.371 to- 0.006  -0.345 to 0.034  -0.275 to -0.080  -0.221 to -0.014 |  | 0.038  0.043  0.109  < 0.001***  0.027 | 0.418  0.473  1.000  < 0.01**  0.297 |
|  |  | Time: PMQ 1  Time: PMQ 2  Time: PMQ 3  Time: PMQ 4  Time: PMQ 5 | -0.044  -0.066  -0.005  -0.130  0.018 | -0.265 to -0.178  -0.257 to 0.126  -0.203 to 0.193  -0.230 to -0.029  -0.084 to 0.120 |  | 0.700  0.503  0.958  0.011*  0.728 | 1.000  1.000  1.000  0.121  1.000 |
|  | Aggressive behavior | Time | 0.107 | -0.010 to 0.225 | 0.029 | 0.074 | 0.814 |
|  |  | PMQ 1  PMQ 2  PMQ 3  PMQ 4  PMQ 5 | -0.276  -0.014  -0.336  -0.258  -0.098 | -0.560 to 0.008  -0.261 to 0.233  -0.593 to -0.079  -0.390 to -0.126  -0.238 to 0.042 |  | 0.057  0.913  0.010  < 0.001***  0.170 | 0.626  1.000  1.000  < 0.01***  1.000 |
|  |  | Time: PMQ 1  Time: PMQ 2  Time: PMQ 3  Time: PMQ 4  Time: PMQ 5 | -0.206  -0.050  0.095  -0.087  -0.034 | -0.470 to 0.059  -0.279 to 0.179  -0.141 to 0.332  -0.207 to 0.033  -0.156 to 0.088 |  | 0.128  0.671  0.429  0.153  0.582 | 1.000  1.000  1.000  1.000  1.000 |
| ****p*<0.001, ***p*<0.01, **p*<0.05  *Note.* PMQ 1, Parental monitoring of location; PMQ 2, Parental monitoring of who their children spend time with, PMQ 3, Parent and child contact; PMQ 4, Child disclosure; PMQ 5, Family dinner frequency. | | | | | | | |

**Table S15. Relationship between Parent Monitoring Behavior Strength and Time Point**

| Outcome | | Variable | B | 95%CI | *R*^2^ | *p* value | |
| --- | --- | --- | --- | --- | --- | --- | --- |
| CBCL | Withdrawn/depressed | 1 SD above the mean of PMQ subitem4: Time | 0.178 | -0.071 to 0.426 | 0.045 | | 0.161 |
|  |  | 1 SD below the mean of PMQ subitem4: Time | 0.557 | 0.315 to 0.800 |  | < 0.001*** | |
|  | Rule-breaking behavior | 1 SD above the mean of PMQ subitem4: Time | -0.148 | -0.294 to -0.003 | 0.034 | 0.046* | |
|  |  | 1 SD below the mean of PMQ subitem4: Time | 0.123 | -0.019 to 0.265 |  | 0.091 | |
| *Note. SD,* standard deviation; CBCL, Child Behavior Checklist; PMQ subitem4, Parental Monitoring Questionnaire Child Disclosure | | | | | | | |

**Figure S2.** **Simple slopes analysis of cross-level interaction of PMQ subitem4 and time point in mental health such as withdrawn/depressed, and rule-breaking behavior**.


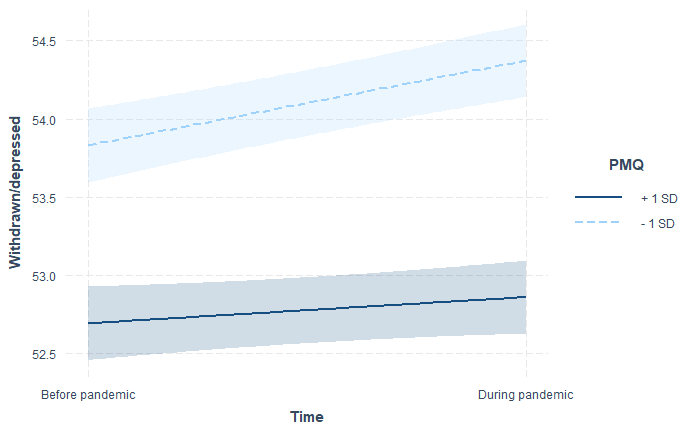


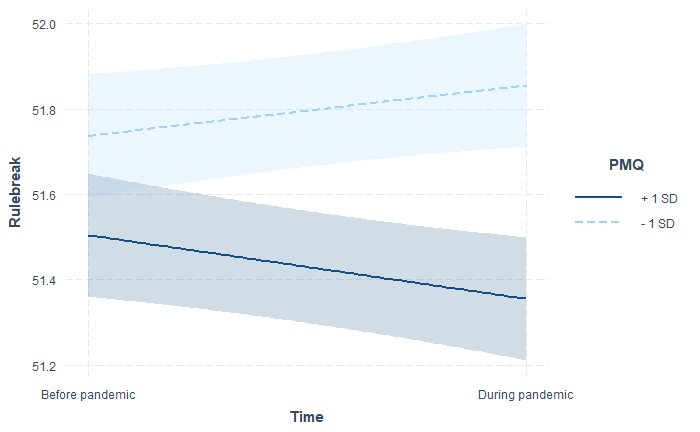


Between the withdrawn/depressed and the time point and the time point showed a significant positive association when PMQ subitem4 was 1 SD below the mean. As well, the rule-breaking behavior and the time point showed a negative association when PMQ subitem4 was 1 SD above the mean. *Notes.* SD: standard deviation; -1SD: 1SD below the mean of PMQ; +1SD: 1SD above the mean of PMQ; PMQ: Parental Monitoring Questionnaire

**Table S16**. **Comparison of baseline in CBCL in the dropout group and the non-dropped group**

|  | Group A | Group B | *p* value |
| --- | --- | --- | --- |
|  | *N =* 5625 | *N =* 6251 |  |
| CBCL (*T*-score) | *Mean* ± *SD* | *Mean* ± *SD* |  |
| Total score | 46.0 ± 11.7 | 45.7 ± 11.0 | 0.2 |
| Internalizing problem | 48.4 ± 10.8 | 48.5 ± 10.5 | >0.9 |
| Externalizing problem | 46.0 ± 10.6 | 45.5 ± 10.1 | 0.003 |
| Withdrawn/depressed | 53.6 ± 5.9 | 53.4 ± 5.7 | 0.066 |
| Somatic complaints | 54.9 ± 6.1 | 54.9 ± 6.0 | >0.9 |
| Social problems | 52.9 ± 4.9 | 52.7 ± 4.6 | 0.004 |
| Thought problems | 53.9 ± 6.2 | 53.7 ± 5.7 | 0.009 |
| Attention problems | 54.1 ± 6.3 | 53.8 ± 6.0 | 0.003 |
| Rule-breaking behavior | 53.1 ± 5.3 | 52.5 ± 4.5 | <0.001 |
| Aggressive behavior | 53.0 ± 5.7 | 52.7 ± 5.3 | 0.004 |
| Anxious/depressed | 53.5 ± 6.1 | 53.4 ± *5*.9 | 0.4 |
| Note. M, mean; SD, standard deviation; CBCL, Child Behavior Checklist; Group A, Group A are dropouts with no follow-up data for the third year; Group B, Group B are people who have follow-up data for the third year.  *Welch Two Sample t-test | | | |
